# Bleomycin-Induced Subcutaneous Fibrosis and Autologous Fat Graft Remodeling Assessed by Ultrasonography in a Rat Experimental Model

**DOI:** 10.3390/bioengineering13040390

**Published:** 2026-03-27

**Authors:** Razvan George Bogdan, Anca Maria Cimpean, Alina Helgiu, Mara Nicolau, Ioan Cătălin Bodea, Rodica Elena Heredea, Zorin Petrisor Crainiceanu

**Affiliations:** 1 Doctoral School, “Victor Babeș” University of Medicine and Pharmacy Timișoara, 300041 Timisoara, Romania; razvan.bogdan@umft.ro; 2 County Clinical Emergency Hospital “Pius Brînzeu”, 300723 Timisoara, Romania; 3 Department of Microscopic Morphology, Histology, “Victor Babeș”, University of Medicine and Pharmacy Timisoara, 300041 Timisoara, Romania; 4 Center of Expertise for Rare Vascular Disease in Children, Emergency Hospital for Children Louis Turcanu, 300011 Timisoara, Romania; 5 Center of Genomic Medicine, “Victor Babeș” University of Medicine and Pharmacy, 300041 Timisoara, Romania; 6 Research Center for Pharmaco-Toxicological Evaluation, “Victor Babes” University of Medicine and Pharmacy, 300041 Timisoara, Romania; 7 Faculty of Medicine, “Lucian Blaga” University of Sibiu, 550024 Sibiu, Romania; 8 County Clinical Emergency Hospital of Sibiu, 550245 Sibiu, Romania; 9 Department of Surgery, “Iuliu Hateganu” University of Medicine and Pharmacy, Croitorilor Street, No. 19-21, 400162 Cluj-Napoca, Romania; 10 “Octavian Fodor” Regional Institute of Gastroenterology and Hepatology, Croitorilor Street, No. 19-21, 400162 Cluj-Napoca, Romania; 11 Discipline of Clinical Practical Skills, Department I Nursing, Faculty of Medicine, “Victor Babeș” University of Medicine and Pharmacy, 300041 Timisoara, Romania; 12 Plastic Surgery Department, “Victor Babeș” University of Medicine and Pharmacy, 300041 Timisoara, Romania

**Keywords:** bleomycin-induced fibrosis, ultrasonography, autologous fat grafting, subcutaneous remodeling, soft tissue engineering, semi-quantitative imaging, regenerative intervention, longitudinal experimental model

## Abstract

Radiation-associated soft tissue fibrosis represents a progressive structural remodeling process characterized by extracellular matrix accumulation, septal thickening, and reduced tissue compliance, which complicates reconstructive interventions. Reliable longitudinal experimental frameworks capable of non-invasive structural monitoring remain limited. This study aimed to develop and implement a standardized ultrasonographic protocol for the evaluation of bleomycin-induced subcutaneous fibrosis and to assess remodeling dynamics following autologous fat grafting in a rat model. Twenty-two adult female Wistar rats were included. Subcutaneous fibrosis was induced using submaximal bleomycin administration (1 mg/kg/day for three consecutive days). High-frequency ultrasonography (12 MHz) was performed at baseline (Day 0), post-bleomycin (Day 17), and post-lipofilling (Day 31). A predefined semi-quantitative 0–3 scoring system was applied to characterize global echogenicity, septal thickening, and architectural organization. At Day 17, all animals demonstrated structural alteration with a mean score of 2.15 ± 0.58. At Day 31, the mean score decreased to 1.50 ± 0.50, with complete disappearance of high-grade architectural disorganization (score 3). Focal hypoechoic areas consistent with graft integration were observed in 88.9% of animals without ultrasonographic signs of necrosis or fluid collection. This standardized imaging-based framework enables reproducible longitudinal monitoring of early-stage subcutaneous fibrotic remodeling and provides a non-invasive tool for evaluating regenerative interventions in translational soft tissue engineering research.

## 1. Introduction

Radiotherapy remains a fundamental component of modern breast cancer management, significantly improving local control and overall survival across disease stages [1,2]. However, exposure of soft tissues to ionizing radiation frequently induces progressive radiation-associated fibrosis, a chronic remodeling process characterized by excessive extracellular matrix deposition, septal thickening, reduced tissue compliance, and architectural disorganization of the subcutaneous compartment [3,4,5]. In the breast and chest wall, this fibrotic transformation manifests clinically as tissue induration, decreased elasticity, contour deformities, and impaired reconstructive outcomes, representing a substantial long-term burden for survivors [6,7]. These structural alterations directly influence tissue biomechanics and regenerative capacity, complicating both aesthetic and functional restoration. Despite advances in oncologic therapies, effective strategies to prevent or reverse established fibrotic remodeling remain limited, partly due to the absence of standardized longitudinal experimental frameworks capable of reproducing clinically relevant early-stage subcutaneous fibrosis and enabling non-invasive structural monitoring over time [3,8]. Controlled preclinical systems that mimic mild yet measurable fibrotic changes are therefore essential for evaluating regenerative interventions in a translationally meaningful manner.

To experimentally reproduce clinically relevant fibrotic remodeling in a controlled setting, bleomycin-induced fibrosis models have been widely employed in preclinical research. Subcutaneous administration of bleomycin in rodents produces progressive extracellular matrix accumulation, septal thickening, and dermal–subcutaneous architectural alterations that resemble key structural features observed in radiation-associated fibrosis [9,10]. These models are valued for their reproducibility, dose-dependent response, and capacity to generate early-stage fibrotic remodeling while preserving overall tissue integrity, allowing subsequent therapeutic intervention [10,11]. Importantly, bleomycin-induced fibrosis has been extensively used to evaluate antifibrotic and regenerative strategies, serving as a translational bridge between mechanistic studies and clinical application [12,13]. While most investigations rely predominantly on terminal histological assessment, longitudinal structural evaluation within the same subject remains limited. In this context, high-frequency ultrasonography represents a practical, non-invasive tool capable of characterizing subcutaneous architecture and enabling comparative pre- and post-intervention assessment in experimental models.

Autologous fat grafting has emerged as a promising regenerative strategy for the management of radiation-induced soft-tissue damage, particularly in the setting of breast reconstruction. Beyond its volumetric role, adipose tissue transplantation has been associated with improvements in tissue pliability, vascularization, and overall structural quality of irradiated skin and subcutaneous tissue [14,15,16]. Clinically, lipofilling has been reported to reduce fibrosis-related induration and enhance reconstructive outcomes, contributing to both functional and aesthetic restoration [15,17]. Experimental studies further suggest that grafted adipose tissue may influence local remodeling processes and partially reverse fibrotic architectural alterations [18]. However, despite increasing clinical application, objective and standardized methods for evaluating structural integration and remodeling dynamics following fat grafting remain limited. In both experimental and clinical contexts, assessment often relies on subjective or semi-quantitative examination, histological endpoints, or indirect imaging parameters, highlighting the need for complementary objective measurements, underscoring the need for reproducible structural monitoring tools capable of capturing pre- and post-intervention changes.

Accurate assessment of fibrotic remodeling remains a central challenge in both experimental and clinical settings. Histological analysis is widely regarded as the reference standard for characterizing extracellular matrix deposition and architectural disruption; however, it is inherently terminal and does not allow repeated evaluation within the same subject over time. Clinical assessment of tissue stiffness and induration is often subjective, while advanced modalities such as elastography or magnetic resonance imaging may be limited by accessibility, cost, or lack of standardization in small-animal models [19,20]. In contrast, high-frequency ultrasonography provides a non-invasive, repeatable, and anatomically precise method for visualizing dermal–subcutaneous structure, including layer thickness, echogenicity patterns, septal organization, and focal structural alterations. Despite its potential, standardized longitudinal ultrasonographic frameworks specifically designed to monitor subcutaneous fibrotic remodeling and post-grafting integration in preclinical models remain insufficiently developed. Establishing reproducible imaging protocols and semi-quantitative evaluation systems is therefore essential to enable objective comparison between baseline, fibrotic, and post-intervention stages.

Despite the growing use of bleomycin-induced fibrosis models and the increasing clinical application of autologous fat grafting in irradiated tissues, a reproducible framework for longitudinal structural monitoring in experimental settings remains lacking. Most preclinical studies evaluate therapeutic outcomes using endpoint histology or indirect surrogate measures, which do not capture dynamic remodeling processes within the same subject. Moreover, standardized ultrasonographic criteria capable of differentiating baseline architecture, early fibrotic alterations, and post-grafting structural changes have not been clearly established for small-animal subcutaneous models. The absence of a semi-quantitative, anatomically consistent imaging protocol limits objective comparison across timepoints and between studies. A structured ultrasonographic evaluation system designed specifically for controlled pre- and post-intervention assessment would therefore represent a meaningful methodological contribution to translational fibrosis research.

The aim of the present study was to develop and implement a standardized longitudinal ultrasonographic protocol for the structural evaluation of bleomycin-induced subcutaneous fibrosis in a rat model and to assess remodeling dynamics following autologous fat grafting. By integrating controlled induction of mild fibrosis, anatomically consistent imaging planes, and a semi-quantitative scoring system designed to characterize subcutaneous architectural features, we sought to enable objective comparison between baseline, fibrotic, and post-intervention stages. This imaging-based framework provides a reproducible and non-invasive approach for monitoring structural remodeling over time within the same subject. By facilitating standardized pre- and post-lipofilling assessment, the proposed methodology may contribute to improved experimental consistency and support future translational studies investigating regenerative strategies in radiation-associated soft-tissue fibrosis.

However, this limitation is partially mitigated in the present study by the inclusion of objective quantitative ultrasonographic parameters, which provide reproducible measurements of tissue structure and support the interpretation of semi-quantitative findings.

## 2. Materials and Methods

### 2.1. Study Design

This study was designed as a controlled, longitudinal experimental investigation aimed at establishing a reproducible model of bleomycin-induced subcutaneous fibrosis and evaluating structural tissue remodeling following autologous fat grafting using high-frequency ultrasonography.

A total of 22 adult female Wistar rats were included according to the approved experimental protocol. Animals were allocated into three predefined groups: a bleomycin + lipofilling group (n = 15), a bleomycin-only fibrosis group (n = 5), and a histological reference group (n = 2). The reference group was euthanized at baseline for tissue harvesting and was not included in longitudinal ultrasonographic analysis.

Ultrasonographic assessment was performed in 20 animals, corresponding to the bleomycin + lipofilling group and the bleomycin-only group.

The experimental unit was the individual animal. Ultrasonographic examinations were conducted longitudinally within the same standardized anterior thoracic region at predefined timepoints.

The total duration of the experimental protocol was 31 days.

Two animals from the treatment group reached predefined humane endpoints due to wound dehiscence and graft extrusion after autologous fat grafting and were excluded from the final longitudinal ultrasonographic assessment. Therefore, ultrasonographic evaluation included 20 animals at baseline (Day 0) and Day 17, and 18 animals at Day 31.

This manuscript exclusively reports the ultrasonographic component of the experimental framework. Clinical and histological analyses derived from the same model are reported separately.

This study was designed as an exploratory experimental investigation aimed primarily at establishing and validating a reproducible ultrasonographic monitoring framework for bleomycin-induced subcutaneous fibrosis and post–fat graft remodeling. Because the primary objective was methodological standardization and longitudinal imaging feasibility rather than hypothesis-driven group comparison, the study was not powered for extensive intergroup statistical comparisons.

Animals were therefore allocated preferentially to the treatment group in order to maximize longitudinal imaging observations after lipofilling. The bleomycin-only group served as a fibrosis control for structural comparison, while the small reference group was used exclusively for baseline histological characterization of normal subcutaneous architecture.

### 2.2. Animals and Housing Conditions

Twenty-two adult female Wistar rats (Rattus norvegicus), 16 weeks of age and weighing 250–300 g at inclusion, were used in this study. Female animals were selected to maintain biological homogeneity within the experimental cohort and to reduce variability associated with sex-dependent differences in body composition and hormonal status.

Animals were housed in a specific pathogen-free facility within the Center for Training and Experimental Surgery “Pius Brînzeu”, Victor Babeș University of Medicine and Pharmacy Timișoara, Romania. A 7-day acclimatization period was observed prior to the initiation of experimental procedures.

Rats were housed in groups of 3–5 per cage under controlled environmental conditions, with ambient temperature maintained at 22 °C and a 12 h light/12 h dark cycle. Standard laboratory chow and water were provided ad libitum. Environmental enrichment included nesting material and cage structural elements. Individual animals were identified using non-invasive tail marking with a permanent marker to allow consistent longitudinal identification during ultrasonographic examinations. This method was selected to minimize stress and avoid procedures that could interfere with animal welfare or experimental outcomes [21].

Animals were housed in a specific pathogen-free facility within the Experimental Surgery and Training Center “Pius Brînzeu”, Timișoara, Romania, operating under veterinary sanitary authorization no. 815/11 June 2021.

All procedures complied with Directive 2010/63/EU and national legislation governing the protection of animals used for scientific purposes. The study protocol was approved by the Research Ethics Committee of the “Victor Babeș” University of Medicine and Pharmacy Timișoara (Approval No. 54/25 November 2022, revised 20 February 2026).

### 2.3. Experimental Timeline

The experimental protocol followed a predefined longitudinal structure over 31 days.

Day 0: Baseline ultrasonographic assessment of the anterior thoracic subcutaneous tissue.

Days 1–3: Subcutaneous administration of bleomycin at a dose of 1 mg/kg body weight per day.

Day 17: Fourteen days after the final bleomycin injection, animals underwent repeat ultrasonographic evaluation of the same predefined anterior thoracic region. Immediately following this assessment, autologous fat grafting was performed in the treatment group.

Day 31: Fourteen days after autologous fat grafting, a final ultrasonographic evaluation was performed in all surviving animals included in the imaging analysis.

All ultrasonographic examinations were performed within the same anatomically standardized anterior thoracic field in each animal.

### 2.4. Induction of Subcutaneous Fibrosis

Subcutaneous fibrosis was induced using bleomycin sulfate (15,000 IU/vial; Accord Healthcare Polska Sp. z o.o., Warsaw, Poland). The lyophilized powder was reconstituted with sterile 0.9% sodium chloride solution to obtain a final concentration of 1 mg/mL.

Bleomycin was administered subcutaneously at a dose of 1 mg/kg body weight per day for three consecutive days (Days 1–3). The injected volume was calculated individually according to each animal’s body weight.

Injections were performed using sterile 1 mL syringes equipped with 26G needles. A single predefined injection point was used within the standardized anterior thoracic region that was subsequently subjected to longitudinal ultrasonographic evaluation. The needle was inserted strictly into the subcutaneous plane to avoid intramuscular administration.

The cumulative bleomycin dose per animal was 3 mg/kg.

This submaximal dosing protocol was selected to induce controlled early-stage subcutaneous fibrotic remodeling while preserving tissue integrity, allowing subsequent evaluation of structural changes following regenerative intervention.

The selected dosing regimen was based on previously published bleomycin-induced skin fibrosis models demonstrating that repeated subcutaneous administration in the range of 0.5–2 mg/kg/day produces progressive dermal and subcutaneous fibrotic remodeling while preserving tissue viability. A dose of 1 mg/kg/day administered for three consecutive days was selected as a submaximal induction protocol aimed at generating early-stage structural fibrosis without inducing extensive tissue necrosis or destructive sclerosis, thereby allowing subsequent evaluation of regenerative intervention.

### 2.5. Autologous Fat Grafting Procedure

Fourteen days after the final bleomycin administration (Day 17), animals underwent surgical intervention under general anesthesia induced by intraperitoneal administration of ketamine (100 mg/kg) and xylazine (10 mg/kg).

The anterior thoracic region previously subjected to bleomycin injection and longitudinal ultrasonographic evaluation was re-identified and prepared under sterile conditions. The thoracic and inguinal areas were shaved and disinfected using 10% povidone–iodine solution.

Subcutaneous adipose tissue was harvested from the inguinal region through a small incision using blunt dissection. Minor vascular branches were coagulated using bipolar electrocautery. The harvested adipose tissue was collected as free, non-vascularized graft material. The term non-vascularized refers to the fact that the adipose tissue was transplanted as a free graft without preservation of an intact vascular pedicle. No intentional stripping of microvascular structures was performed beyond standard surgical harvesting.

The tissue was rinsed with sterile saline and mechanically fragmented without enzymatic processing. Mechanical fragmentation was performed manually using sterile surgical scissors until small adipose fragments of approximately 1–2 mm were obtained. No enzymatic digestion or centrifugation was used in order to preserve the structural integrity of the grafted adipose clusters. The processed adipose tissue was transferred into a sterile 1 mL syringe.

At the predefined anterior thoracic site, corresponding to the ultrasonographic field of evaluation, a minimal subcutaneous recipient pocket was created through a small incision. A volume of 0.5 ± 0.08 mL of fragmented adipose tissue was injected within a single subcutaneous plane using a micro-deposit technique, avoiding intramuscular placement and avoiding creation of an extensive artificial cavity. The graft was delivered using a controlled low-pressure injection technique within the same subcutaneous plane in order to avoid the formation of a large compact bolus and to facilitate the distribution of the adipose fragments within the recipient compartment.

The injection plane corresponded strictly to the previously imaged subcutaneous compartment to ensure anatomical consistency between baseline, post-bleomycin, and post-grafting ultrasonographic assessments.

Donor and recipient sites were closed using 4-0 polypropylene monofilament sutures.

### 2.6. Ultrasonographic Assessment Protocol

Ultrasonographic examinations were performed using a Vscan Air™ portable ultrasound system equipped with a 12 MHz linear probe (GE HealthCare, Chicago, IL, USA).

All assessments were conducted within the same predefined anterior thoracic anatomical region corresponding to the bleomycin injection site and subsequent graft placement.

At baseline (Day 0), ultrasonographic evaluation was performed without anesthesia under gentle manual restraint in dorsal recumbency. At Day 17 and Day 31, examinations were performed under general anesthesia induced with ketamine (100 mg/kg) and xylazine (10 mg/kg) to minimize motion artifacts and ensure reproducible probe positioning. Because baseline ultrasonographic evaluation did not involve surgical manipulation, anesthesia was not used at Day 0 in order to avoid unnecessary pharmacological exposure. Subsequent examinations were performed under anesthesia to ensure stable positioning following surgical intervention and to minimize motion artifacts during post-intervention imaging.

Animals were positioned in dorsal recumbency. Acoustic gel was applied to the shaved anterior thoracic field. The linear probe was placed perpendicular to the skin surface with minimal pressure to avoid artificial compression of the subcutaneous layer.

Imaging parameters, including gain, imaging depth, and focal zone, were maintained constant throughout all examinations to ensure longitudinal comparability.

The subcutaneous compartment was evaluated in B-mode. All examinations were digitally recorded to allow subsequent review and standardized interpretation.

Image analysis was performed offline by an experienced soft-tissue ultrasonographer who was familiar with small-animal imaging. Evaluation focused on dermal–subcutaneous thickness, echogenicity pattern, structural homogeneity, and identification of graft-related hypoechoic areas at post-intervention timepoints. All images were exported in a standardized format and analyzed offline for both semi-quantitative scoring and quantitative image processing.

### 2.7. Semi-Quantitative Ultrasonographic Evaluation

All ultrasonographic examinations were digitally recorded and subsequently reviewed in their entirety for each animal and each time point.

Image interpretation was performed offline by a single experienced radiologist specialized in soft-tissue ultrasonography. The evaluator was aware of the longitudinal design of the study but assessed each time point independently to ensure consistent structural interpretation.

Ultrasonographic evaluation focused on qualitative structural changes within the dermal–subcutaneous compartment, including global echogenicity, tissue homogeneity, septal thickening, architectural organization, and the presence of focal hypoechoic areas corresponding to grafted adipose tissue at post-intervention timepoints.

A semi-quantitative 0–3 scoring system was applied to classify the degree of subcutaneous structural alteration:

0—Homogeneous, predominantly hypoechoic subcutaneous layer with fine echogenic septa and preserved architecture.

1—Mild increase in echogenicity with subtle septal accentuation and minimal structural heterogeneity.

2—Moderate increase in echogenicity with visible septal thickening and heterogeneous subcutaneous architecture.

3—Marked diffuse hyperechogenicity, pronounced septal thickening, and evident architectural disorganization consistent with advanced fibrotic remodeling.

For animals undergoing autologous fat grafting, focal hypoechoic regions within the subcutaneous plane were documented descriptively at Day 31 as indicators of graft presence and structural integration.

In cases of uncertainty between two adjacent grades, the higher score was assigned.

A single score was recorded for each animal at baseline (Day 0), post-bleomycin reassessment (Day 17), and final evaluation (Day 31).

The evaluator was aware of the longitudinal experimental design but analyzed each time point independently using predefined structural criteria in order to ensure internal consistency of scoring. Because the primary objective of the study was to establish a standardized ultrasonographic framework for structural monitoring, scoring was performed by a single trained evaluator to maintain methodological consistency across all examinations.

The proposed semi-quantitative scoring system was developed to provide a structured descriptive framework for characterizing global architectural alterations of the dermal–subcutaneous compartment in this experimental setting. The score was designed to capture progressive changes in echogenicity, septal prominence, and structural organization observed during fibrotic remodeling. Because the primary objective of the present work was to establish a reproducible imaging protocol for longitudinal monitoring, the scoring system should be considered a structured semi-quantitative classification tool complemented by objective quantitative parameters rather than a fully validated quantitative fibrosis metric.

### 2.8. Quantitative Image Analysis

In addition to the semi-quantitative ultrasonographic assessment, objective measurements were performed using both on-device calipers and offline image analysis.

Dermal and subcutaneous thickness measurements were obtained on the ultrasound device using electronic calipers placed perpendicular to the skin surface at standardized anatomical locations. For each image, five independent measurements were recorded and averaged to reduce local variability.

For extended quantitative analysis, stored B-mode ultrasonographic images were exported and processed using ImageJ software (version 1.53t, National Institutes of Health, Bethesda, MD, USA).

All analyses were conducted under standardized imaging conditions, including constant gain, depth, and probe positioning across all timepoints.

Grayscale analysis was performed to quantify echogenicity and structural heterogeneity. Regions of interest (ROIs) were manually selected within the dermal and subcutaneous layers, avoiding boundary artifacts and large vessels. Mean grayscale intensity was recorded as a measure of echogenicity, while the standard deviation of grayscale values was used as an index of structural heterogeneity. Linear measurements were used to assess dermal and subcutaneous thickness, while rectangular regions of interest were applied for grayscale-based analysis of echogenicity and heterogeneity within each layer.

Relative echogenicity between dermal and subcutaneous layers was considered during analysis as an additional parameter reflecting the proportional relationship between tissue compartments.

Multiple ROIs were analyzed for each layer and time point, and mean values were calculated for statistical evaluation. ROI size was kept consistent within each tissue layer to minimize measurement bias.

All measurements were performed by a single evaluator to ensure methodological consistency. For each animal and time point, multiple representative ultrasonographic images were analyzed, and mean values were calculated to reduce sampling bias. Regions of interest were selected from comparable anatomical locations across all timepoints to ensure longitudinal consistency of measurements. ROI placement avoided extreme values, acoustic artifacts, and non-representative structures in order to provide an objective assessment of tissue characteristics.

The same measurement protocol was applied uniformly across all animals and timepoints to allow direct comparison of quantitative parameters. Quantitative parameters were subsequently used for comparative quantitative evaluation across timepoints.

### 2.9. Statistical Analysis

Given the ordinal nature of the semi-quantitative scores and the longitudinal repeated-measures design, non-parametric statistical analysis was performed. Differences across the three predefined time points (Day 0, Day 17, Day 31) were assessed using the Friedman test. Pairwise comparisons were subsequently performed using the Wilcoxon signed-rank test. Statistical significance was defined as *p* < 0.05.

Due to the exploratory nature of the study and the ordinal structure of the semi-quantitative scoring system, statistical analysis focused on within-subject longitudinal comparisons rather than extensive intergroup hypothesis testing. The sample size was therefore considered sufficient for detecting major structural changes across timepoints within the same animals but not for detecting subtle subgroup effects.

### 2.10. Data Availability

The datasets generated and analyzed during the current study are available from the corresponding author upon reasonable request. No large-scale datasets were deposited in publicly accessible repositories.

## 3. Results

A total of 22 animals were initially included in the experimental protocol. Ultrasonographic assessment was performed in 20 animals at baseline and Day 17. Two animals from the treatment group were excluded after Day 17 due to wound dehiscence and graft extrusion according to predefined humane endpoints. Final ultrasonographic evaluation at Day 31 included 18 animals.

### 3.1. Baseline Ultrasonographic Findings

At baseline (Day 0), ultrasonographic evaluation was performed in 20 animals.

All examinations demonstrated preserved dermal–subcutaneous architecture. The dermal interface appeared continuous and regular. The subcutaneous layer was predominantly hypoechoic, with thin, regularly distributed echogenic septa. No septal thickening, diffuse hyperechogenicity, focal hypoechoic areas, or architectural disorganization were observed.

According to the predefined semi-quantitative scoring system:

- 20/20 animals received a score of 0;
- No animal demonstrated increased echogenicity or septal accentuation;
- Mean baseline score was 0.00 (SD 0).

These findings confirm normal subcutaneous architecture prior to experimental induction of fibrosis.

### 3.2. Ultrasonographic Changes After Bleomycin Administration (Day 17)

At Day 17, ultrasonographic evaluation was performed in 20 animals.

Compared to baseline, all animals demonstrated structural alterations within the subcutaneous compartment. The subcutaneous layer exhibited increased global echogenicity, visible septal thickening, and reduced architectural homogeneity. The dermal interface remained continuous, and the muscular fascia remained identifiable in all cases. No anechoic collections, cavitary lesions, or ultrasonographic signs of tissue necrosis were observed.

According to the predefined semi-quantitative scoring system:

- In total, 2/20 animals (10%) received a score of 1;
- In total, 13/20 animals (65%) received a score of 2;
- In total, 5/20 animals (25%) received a score of 3;
- Mean semi-quantitative score was 2.15 ± 0.58.

These findings confirm consistent induction of early-stage subcutaneous fibrotic remodeling following submaximal bleomycin administration.

### 3.3. Ultrasonographic Findings After Autologous Fat Grafting (Day 31)

At Day 31, ultrasonographic evaluation was completed in 18 animals.

Compared to the post-bleomycin timepoint, global subcutaneous echogenicity was reduced, septal thickening was less pronounced, and architectural heterogeneity was attenuated. No animal maintained a score of 3.

According to the semi-quantitative scoring system:

- In total, 9/18 animals (50%) received a score of 1;
- In total, 9/18 animals (50%) received a score of 2;
- No animal received a score of 3;
- Mean semi-quantitative score was 1.50 ± 0.50.

**Figure 1:**
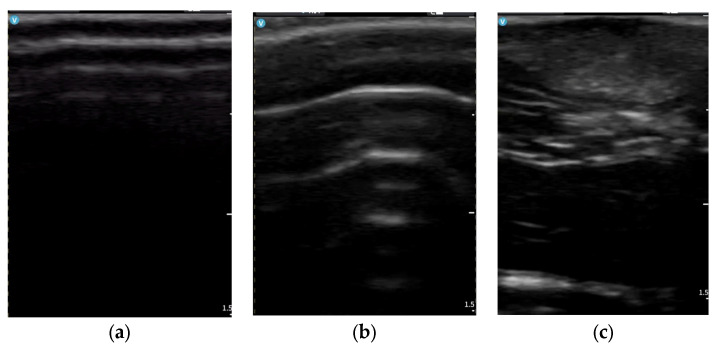
Representative longitudinal ultrasonographic images of the same anatomical region across timepoints. (**a**) Baseline (Day 0): preserved dermal–subcutaneous architecture with a homogeneous hypoechoic subcutaneous layer and fine echogenic septa. (**b**) Day 17: increased echogenicity, septal thickening, and reduced structural homogeneity consistent with early fibrotic remodeling. (**c**) Day 31: partial attenuation of echogenicity and septal prominence with the presence of focal hypoechoic areas corresponding to graft integration.

Focal hypoechoic areas within the subcutaneous plane were identified in 16/18 animals (88.9%), consistent with graft presence and structural integration. Representative longitudinal ultrasonographic images illustrating these structural changes across timepoints are shown in Figure 1. These areas demonstrated partial incorporation within the subcutaneous architecture without evidence of cavitation or fluid collection.

In a subset of animals, larger focal hypoechoic areas were observed, indicating more voluminous graft integration. In the largest observed case, the maximal vertical thickness of the focal hypoechoic graft-related area was approximately 3–4 mm, estimated using the on-screen 5 mm depth calibration.

These areas remained structurally incorporated within the subcutaneous plane and were not associated with fluid collections, internal debris, posterior acoustic enhancement, or other ultrasonographic signs suggestive of fat necrosis or abscess formation. No anechoic collections, internal echogenic debris, posterior acoustic enhancement, encapsulated hypoechoic cavities, or ultrasonographic patterns suggestive of liquefaction or fat necrosis were identified at any time point.

These findings document attenuation of early-stage fibrotic remodeling and ultrasonographic evidence of structural integration of autologous fat graft material.

### 3.4. Longitudinal Evolution of Semi-Quantitative Scores

A progressive change in semi-quantitative ultrasonographic scores was observed across the three predefined timepoints.

At baseline (Day 0), all animals (20/20) demonstrated preserved subcutaneous architecture, with a mean score of 0.00 ± 0.

Following bleomycin administration (Day 17), all examined animals (20/20) showed structural alteration, with scores ranging between 1 and 3 and a mean score of 2.15 ± 0.58.

**Figure 2:**
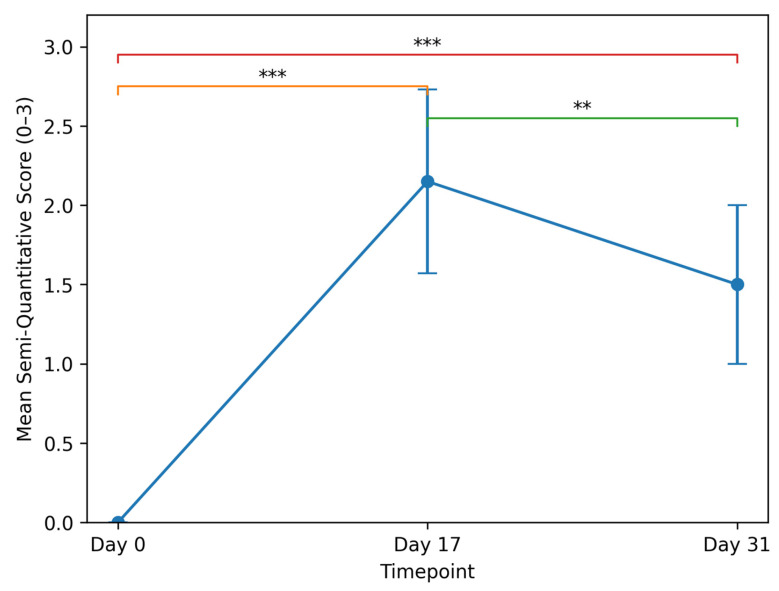
Longitudinal evolution of mean semi-quantitative ultrasonographic scores. Mean structural scores are shown at baseline (Day 0), after bleomycin-induced remodeling (Day 17), and following autologous fat grafting (Day 31). Error bars represent standard deviation (SD). Statistical significance between timepoints is indicated as follows: *** *p* < 0.001, ** *p* < 0.01.

At the final evaluation (Day 31), performed in 18 animals, scores ranged between 1 and 2, with a mean score of 1.50 ± 0.50 (Figure 2). Notably, high-grade architectural disorganization (score 3), observed in 25% of animals at Day 17, was no longer present at the final evaluation. No animal maintained a diffuse hyperechogenic pattern with marked septal thickening at Day 31. The detailed longitudinal distribution of semi-quantitative ultrasonographic scores across all timepoints is summarized in Table 1.

The longitudinal distribution demonstrates:

- Induction of consistent early-stage fibrotic remodeling after bleomycin exposure;
- Absence of complete structural normalization at Day 31;
- Reduction in the proportion of higher-grade alterations after autologous fat grafting;
- Elimination of score 3 cases at final evaluation.

Non-parametric repeated-measures analysis demonstrated a statistically significant difference in semi-quantitative scores across the three timepoints (Friedman test, χ^2^ = 33.63, *p* < 0.001). Pairwise analysis confirmed significant score increase from Day 0 to Day 17 (*p* < 0.001), significant reduction from Day 17 to Day 31 (*p* = 0.002), and persistent difference between Day 0 and Day 31 (*p* < 0.001).

These findings document reproducible induction of early-stage subcutaneous fibrotic remodeling and partial structural improvement following regenerative intervention.

### 3.5. Structural Pattern Characteristics Across Timepoints

At baseline (Day 0), the subcutaneous compartment demonstrated a predominantly hypoechoic pattern with fine, regularly distributed echogenic septa and preserved fascial delineation.

Following bleomycin administration (Day 17), a diffuse shift toward increased echogenicity was observed, characterized by septal accentuation, reduced architectural homogeneity, and global structural densification without fascial disruption.

At the final evaluation (Day 31), the subcutaneous pattern evolved toward a mixed echogenic configuration, with attenuation of septal prominence and partial restoration of architectural uniformity. Focal hypoechoic areas corresponding to graft integration were incorporated within the septal framework without evidence of cavitation or structural collapse.

The dermal–subcutaneous interface remained clearly identifiable at baseline, while partial blurring of layer boundaries was observed at Day 17, consistent with structural disorganization. At Day 31, partial restoration of interface definition was noted in association with reduced echogenicity and septal prominence.

### 3.6. Quantitative Ultrasonographic Analysis

In addition to the semi-quantitative assessment, objective ultrasonographic measurements were performed to characterize structural changes across timepoints.

Dermal thickness increased from 0.94 mm at baseline (Day 0) to 1.39 mm at Day 17, followed by a decrease to 0.79 mm at Day 31.

**Figure 3:**
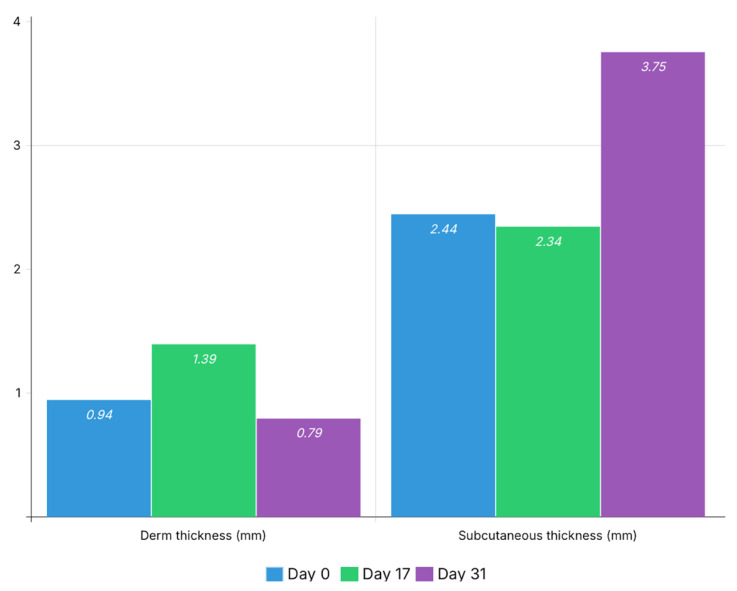
Longitudinal changes in dermal and subcutaneous thickness. Mean thickness values (mm) are presented for each time point (Day 0, Day 17, and Day 31). An increase in dermal thickness is observed at Day 17, followed by a decrease at Day 31, while subcutaneous thickness demonstrates a marked increase at the final timepoint, consistent with graft presence and integration.

Subcutaneous thickness remained relatively stable between baseline and Day 17 (2.44 mm vs. 2.34 mm), followed by a marked increase at Day 31 (3.75 mm). These thickness changes are illustrated in Figure 3.

Grayscale analysis demonstrated a clear increase in dermal echogenicity at Day 17 (30.34 vs. 23.59 at baseline), followed by a decrease at Day 31 (15.42), approaching baseline values.

**Figure 4:**
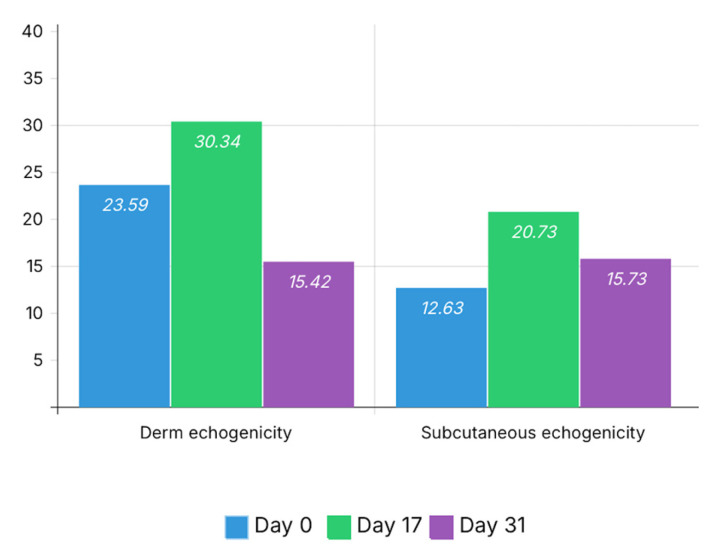
Longitudinal changes in dermal and subcutaneous echogenicity. Mean echogenicity values are presented for each time point (Day 0, Day 17, and Day 31). An increase in echogenicity is observed at Day 17 in both dermal and subcutaneous layers, consistent with fibrotic remodeling, followed by a decrease at Day 31, suggesting partial attenuation of structural alterations after autologous fat grafting.

A similar pattern was observed in the subcutaneous layer, where echogenicity increased from 12.63 at baseline to 20.73 at Day 17, followed by a decrease to 15.73 at Day 31. These echogenicity changes are illustrated in Figure 4.

Heterogeneity indices showed an increase after bleomycin-induced remodeling. Dermal heterogeneity increased from 9.35 at baseline to 14.00 at Day 17, then decreased to 9.70 at Day 31.

**Figure 5:**
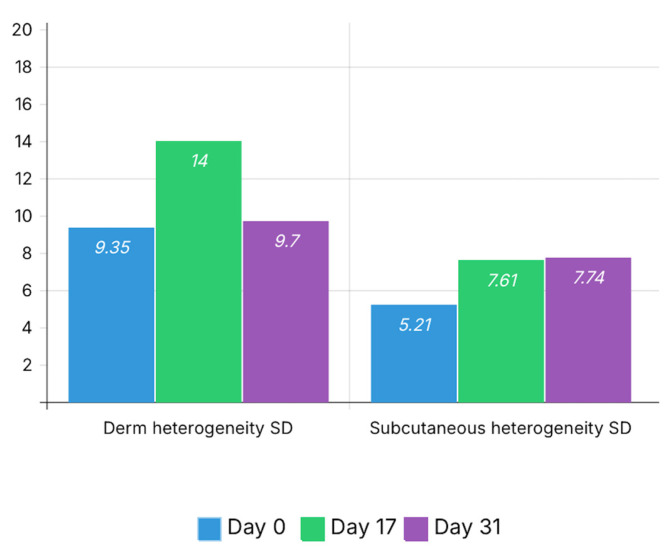
Longitudinal changes in dermal and subcutaneous heterogeneity. Heterogeneity is expressed as the standard deviation (SD) of grayscale intensity within the region of interest for each tissue layer at each time point (Day 0, Day 17, and Day 31). An increase in heterogeneity is observed at Day 17 in both dermal and subcutaneous compartments, reflecting structural disorganization associated with fibrotic remodeling. At Day 31, dermal heterogeneity decreases toward baseline values, while subcutaneous heterogeneity remains moderately elevated, suggesting ongoing structural remodeling following autologous fat grafting.

Subcutaneous heterogeneity increased from 5.21 at baseline to 7.61 at Day 17 and remained elevated at Day 31 (7.74). These heterogeneity changes are illustrated in Figure 5.

These quantitative findings support the semi-quantitative analysis and confirm the presence of fibrotic remodeling at Day 17 and partial structural normalization following autologous fat grafting. All quantitative values are summarized in Table 2.

The quantitative ultrasonographic parameters demonstrated a consistent structural pattern across timepoints that paralleled the semi-quantitative assessment. The increase in dermal and subcutaneous echogenicity observed at Day 17 was accompanied by higher heterogeneity indices, reflecting structural disorganization and septal thickening consistent with early fibrotic remodeling. These changes corresponded to the predominance of semi-quantitative scores of 2 and 3 at this time point.

At Day 31, a reduction in echogenicity and partial normalization of heterogeneity values were observed, particularly at the dermal level. These changes were associated with a shift toward lower semi-quantitative scores, indicating attenuation of structural alterations.

The increase in subcutaneous thickness observed at the final timepoint was associated with the presence of focal hypoechoic graft-related areas, suggesting structural incorporation of adipose tissue within the subcutaneous compartment rather than simple fibrotic expansion.

Overall, the combined evaluation of echogenicity, heterogeneity, and thickness provides a consistent structural profile of fibrotic remodeling and post-intervention changes, supporting the internal consistency of the proposed ultrasonographic evaluation framework.

The observed differences between dermal and subcutaneous echogenicity across timepoints suggest dynamic changes in relative echogenicity between tissue layers, reflecting differential structural involvement of dermal and subcutaneous compartments during fibrotic remodeling and post-intervention reorganization.

## 4. Discussion

Within this experimental framework, the ultrasonographic data provide insight into both the reproducibility of early fibrotic induction and the structural dynamics observed after autologous fat grafting. The selected submaximal bleomycin regimen (1 mg/kg/day for three consecutive days) was intentionally designed to induce controlled early-stage subcutaneous fibrotic remodeling rather than advanced destructive sclerosis. Ultrasonographically, this was reflected by diffuse septal thickening and increased global echogenicity without architectural collapse, fascial disruption, cavitation, or necrotic features. The mean semi-quantitative score of 2.15 at Day 17 indicates reproducible induction of early fibrotic changes across the cohort, with limited inter-individual variability. Importantly, the absence of compact hyperechogenic plates or structural contraction suggests preservation of tissue plasticity. This intermediate fibrotic state closely resembles early radiation-associated subcutaneous alterations and provides an appropriate biological substrate for evaluating the regenerative potential of autologous fat grafting.

The quantitative ultrasonographic analysis further supports these observations by providing objective measurements of structural remodeling across timepoints. Dermal thickness demonstrated a transient increase at Day 17, consistent with early fibrotic changes, followed by a decrease at Day 31, suggesting partial structural normalization. Similarly, dermal echogenicity and heterogeneity increased after bleomycin administration and subsequently decreased following autologous fat grafting, indicating attenuation of fibrotic tissue characteristics.

At the subcutaneous level, echogenicity and heterogeneity increased at Day 17, reflecting structural disorganization, and remained moderately elevated at Day 31, suggesting ongoing remodeling rather than complete normalization. The increase in subcutaneous thickness observed at the final time point may reflect graft presence and integration within the recipient tissue.

Together, these objective parameters complement the semi-quantitative scoring system and support the interpretation of dynamic tissue remodeling rather than static structural alteration.

These findings highlight the added value of integrating objective ultrasonographic parameters with semi-quantitative scoring, as their parallel evolution supports a consistent interpretation of structural remodeling dynamics.

Changes in the definition of the dermal–subcutaneous interface further supported the observed structural evolution, with transient boundary blurring during fibrotic remodeling and partial restoration following autologous fat grafting.

Relative echogenicity between dermal and subcutaneous layers represents an additional informative parameter that may enhance the structural interpretation of ultrasonographic findings. Although not formally quantified in the present study, the observed differential evolution of echogenicity between layers suggests the potential utility of this parameter for future investigations.

The reduction in the mean semi-quantitative score from 2.15 at Day 17 to 1.50 at Day 31 suggests attenuation of early fibrotic architectural alterations following autologous fat grafting. Ultrasonographically, this was characterized by decreased global echogenicity, reduced septal prominence, and partial restoration of subcutaneous architectural homogeneity. Importantly, no animal maintained a score of 3 at final evaluation, indicating the absence of persistent high-grade structural alteration. These findings are compatible with the hypothesis that autologous adipose tissue may exert a modulatory effect on early fibrotic tissue remodeling, promoting structural reorganization rather than merely occupying space within the subcutaneous compartment. Because the present analysis is based exclusively on ultrasonographic findings, these structural changes should be interpreted as imaging indicators of tissue remodeling rather than direct histological confirmation of fibrosis regression.

Preclinical investigations exploring autologous fat grafting in fibrotic environments have suggested antifibrotic and remodeling effects mediated by stromal vascular fraction components and adipose-derived signaling pathways [14,15]. Although most reports rely primarily on histological endpoints, emerging imaging-based studies indicate that regenerative interventions may be accompanied by attenuation of septal thickening and partial restoration of tissue homogeneity [18]. The reduction in semi-quantitative ultrasonographic scores observed in our cohort is consistent with these experimental observations, supporting the concept that adipose tissue grafting may influence early-stage fibrotic remodeling beyond volumetric replacement alone [14,15,18].

The focal hypoechoic regions identified at Day 31 were interpreted as compatible with graft presence and structural incorporation within the subcutaneous compartment. Importantly, these areas were not associated with posterior acoustic enhancement, internal echogenic debris, encapsulated fluid collections, or cavitary patterns that would suggest liquefaction, fat necrosis, or abscess formation. The preservation of fascial continuity and absence of architectural collapse further support their integration within the existing septal framework rather than representing pathological fluid accumulation.

Previous experimental and clinical imaging studies have described early grafted adipose tissue as relatively hypoechoic compared to surrounding fibrotic or septal structures, particularly during the integration phase before complete remodeling of the extracellular matrix [15,18,22]. The structural stability and absence of progressive cavitary transformation in our cohort are consistent with these reports. Nevertheless, in the absence of concurrent histological correlation within the present manuscript, these findings should be interpreted as imaging-compatible indicators of graft integration rather than definitive proof of long-term adipocyte viability [22].

Beyond its experimental relevance, the present model highlights the utility of structured ultrasonographic assessment as a non-invasive tool for longitudinal monitoring of fibrotic remodeling and regenerative intervention. Unlike purely histological endpoints, which require tissue harvesting and preclude dynamic follow-up within the same subject, imaging-based evaluation allows repeated structural assessment over time while preserving anatomical continuity. This characteristic enhances the translational value of the model, particularly in the context of preclinical testing of antifibrotic or cell-based therapies. From a clinical perspective, the ability to detect modulation of septal organization and subcutaneous architecture through standardized ultrasonographic criteria parallels current trends toward imaging-guided evaluation of tissue quality after lipofilling. While direct extrapolation to human post-radiotherapy tissue must be approached cautiously, the present findings support the feasibility of integrating structured imaging parameters into regenerative research pipelines, bridging experimental and clinical practice.

Several limitations should be acknowledged when interpreting the present findings. First, the ultrasonographic evaluation relied on a semi-quantitative scoring system applied by a single trained evaluator, which inherently introduces a degree of observer dependency despite the use of predefined criteria. Second, although non-parametric inferential analysis was performed, the study was not powered for extensive subgroup comparisons. Third, although the structural changes observed were consistent with graft integration and remodeling attenuation, the present manuscript does not include concurrent histological correlation, which will be reported separately. Finally, while the submaximal bleomycin protocol was intentionally selected to model early-stage remodeling, this approach may limit direct comparability with studies employing more aggressive fibrotic induction paradigms. Additionally, the follow-up period is limited to Day 31, which reflects early structural remodeling and does not allow evaluation of long-term graft survival or late-stage fibrotic evolution.

Future investigations integrating quantitative ultrasonographic measurements such as elastography or backscatter-based analysis, with histological and molecular analyses derived from the same experimental framework, will allow deeper mechanistic correlation between imaging findings and tissue-level remodeling [23]. Validation of the proposed semi-quantitative scoring system in independent cohorts and across different fibrosis induction intensities may further refine its reproducibility and applicability. In addition, incorporation of objective imaging metrics, such as thickness measurements or texture-based analysis, could enhance sensitivity in detecting subtle structural modulation following regenerative intervention.

In addition, Doppler-based ultrasonographic techniques may provide complementary information regarding vascular perfusion and neovascularization within the grafted and fibrotic tissue. Although not included in the present study, integration of perfusion imaging could further enhance the functional interpretation of structural remodeling in future experimental models.

Another important limitation relates to the relatively small and imbalanced group sizes, particularly the small histological reference group. The primary objective of the present work was to establish a reproducible imaging framework for longitudinal monitoring rather than to perform statistically powered intergroup comparisons. Consequently, a larger proportion of animals was intentionally allocated to the lipofilling group in order to maximize post-intervention imaging observations. The reference group was used solely to confirm baseline histological architecture and was not intended for formal statistical comparison. The relatively small cohort may also contribute to increased variability in the distribution of semi-quantitative scores across timepoints.

The absence of blinded multi-observer evaluation represents another limitation. Ultrasonographic interpretation was performed by a single experienced evaluator who was aware of the experimental design. While predefined structural criteria were applied consistently across all examinations, the absence of independent blinded raters may introduce observer-related bias. Future studies validating the proposed semi-quantitative scoring framework should incorporate blinded multi-observer assessment and inter-observer reliability analysis. Additionally, integration of objective quantitative parameters such as subcutaneous thickness measurements or texture-based imaging analysis could further strengthen the reproducibility of this imaging approach.

Another methodological consideration relates to the semi-quantitative ultrasonographic scoring system used to characterize subcutaneous structural alterations. Although the scoring framework allowed consistent longitudinal characterization of architectural changes across timepoints, it has not yet undergone formal validation in experimental fibrosis models. The score was designed as a structured descriptive tool capturing global echogenicity, septal thickening, and architectural organization rather than as a direct quantitative measure of fibrosis severity. Future studies integrating histological correlation and objective imaging parameters will be required to validate the proposed scoring system and determine its sensitivity for detecting subtle remodeling dynamics.

The inclusion of quantitative ultrasonographic parameters in the present analysis partially addresses the limitations associated with subjective semi-quantitative evaluation by providing objective, reproducible measurements of tissue structure.

An additional methodological consideration relates to the use of different anesthesia conditions between baseline and follow-up examinations. Although probe positioning and imaging parameters were carefully standardized, the absence of anesthesia at baseline and its use at subsequent timepoints may represent a potential source of variability in ultrasonographic appearance.

Despite these limitations, the integration of standardized quantitative ultrasonographic parameters strengthens the reproducibility and interpretability of the imaging findings.

## 5. Conclusions

This study demonstrates that a controlled submaximal bleomycin protocol reliably induces reproducible early-stage subcutaneous fibrotic remodeling detectable through structured ultrasonographic assessment. The observed attenuation of semi-quantitative ultrasonographic scores following autologous fat grafting suggests structural changes compatible with attenuation of early fibrotic architecture rather than simple volumetric replacement. The elimination of high-grade structural alterations at final evaluation is compatible with structural remodeling processes within a preserved anatomical framework.

Together, these findings validate the proposed imaging-based model as a reproducible platform for longitudinal evaluation of regenerative interventions targeting early fibrotic tissue remodeling.

## Figures and Tables

**Table 1:** Longitudinal distribution of semi-quantitative ultrasonographic scores.

Timepoint	n	Score 0	Score 1	Score 2	Score 3	Mean ± SD
Day 0	20	20 (100%)	0	0	0	0.00 ± 0
Day 17	20	0	2 (10%)	13 (65%)	5 (25%)	2.15 ± 0.58
Day 31	18	0	9 (50%)	9 (50%)	0	1.50 ± 0.50

**Table 2:** Quantitative ultrasonographic parameters across timepoints. Values represent mean measurements obtained from multiple regions of interest per time point.

Parameter	Day 0	Day 17	Day 31
Derm thickness (mm)	0.94	1.39	0.79
Subcutaneous thickness (mm)	2.44	2.34	3.75
Derm echogenicity	23.59	30.34	15.42
Derm heterogeneity (SD)	9.35	14.00	9.70
Subcutaneous echogenicity	12.63	20.73	15.73
Subcutaneous heterogeneity (SD)	5.21	7.61	7.74

## Data Availability

The datasets generated and analyzed during the current study are available from the corresponding author upon reasonable request.

## Abbreviations

The following abbreviations are used in this manuscript:

IU	International units
G	Gauge
SD	Standard deviation
EU	European Union
